# Symplastic scrotal leiomyoma: a case report

**DOI:** 10.1186/1752-1947-2-295

**Published:** 2008-09-09

**Authors:** Joe Philip, Ramaswamy Manikandan, Palaniswamy Vishwanathan, Joseph Mathew

**Affiliations:** 1 Department of Urology, Royal Cornwall Hospital, Truro, TR1 3LJ, UK; 2 Department of Urology, Royal Liverpool University Hospital, Liverpool, L7 8XP, UK; 3 Department of Pathology, Royal Cornwall Hospital, Truro, TR1 3LJ, UK

## Abstract

**Introduction:**

Scrotal leiomyomas are rare tumours which are essentially benign. Recurrence and malignant transformation to leiomyosarcoma have been reported. However, a specific subgroup with increased bizarre nuclei showing increased mitosis raises the need for a closer follow-up. We report on such a case.

**Case presentation:**

We report the case of a 65-year-old man who underwent a scrotal lump excision. Histology showed a well defined leiomyoma. The presence of nuclear pleomorphism and mitoses, just falling short of the criteria for malignancy, made prediction of biological behaviour difficult. The patient remains well on 4-year follow-up.

**Conclusion:**

Histological evidence of increased mitosis raises the need for sustained follow-up in view of the malignant potential from the extent of mitosis. Immunohistochemistry helps in identifying those patients warranting close follow-up.

## Introduction

Leiomyoma of the scrotum is a rare entity described as a benign pathology. Immunohistochemistry helps differentiate this condition from a leiomyosarcoma. However, we raise the entity of symplastic scrotal leiomyoma with bizarre nuclei and increased mitosis on immunohistochemistry. The pattern of growth in this distinct subset is not known. Theoretically, there is a higher risk for malignant transformation. We discuss this situation and suggest the necessity for close follow-up.

## Case presentation

**Figure 1:**
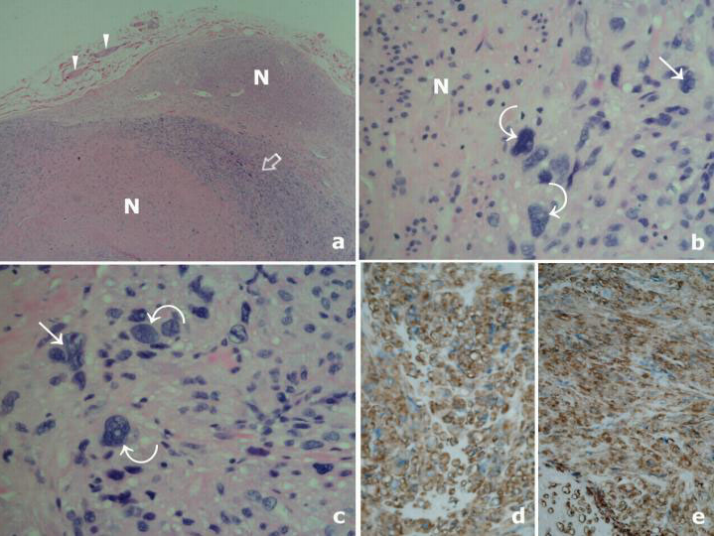
Histology shows: (a) A well-defined lesion, with a pseudocapsule, made up of interlacing bundles of regular smooth muscle cells (N) with apparent hypercellular areas (open arrow); residual bundles of dartoic muscle are seen in adjacent fibrovascular connective tissue (arrow heads) (7.5×; haematoxylin and eosin). (b) & (c) Degenerating uninucleate (curved arrows) and multinucleate giant cells (straight arrow) are seen adjacent to typical leiomyomatous areas (N) (120×; haematoxylin and eosin). (d) Tumour is smooth muscle actin-positive (30×; immunoperoxidase stain, diaminobenzidine method). (e) Tumour is also desmin-positive (30×; immunoperoxidase stain, diaminobenzidine method).

A 65-year-old man presented with a single well-defined, soft, non-tender, mobile right scrotal lump, increasing in size for 4 weeks, with no palpable connection to his testis, epididymis or spermatic cord. Herniae and palpable inguinal lymph nodes were absent. An ultrasound of the testes as well as testicular tumour markers were not undertaken as the testes were considered normal on clinical examination with the lesion being a testicular adnexal mass. The lump was excised from the scrotal dartos layer. Histology showed a well-defined leiomyoma made up of spindle cells in which numerous degenerating uni- and multinucleate tumour giant cells (symplastic, bizarre) were identified; nuclear pleomorphism and four mitoses/ten high power fields were also noted. The tumour was smooth muscle actin- and desmin-positive, confirming smooth muscle phenotype (Fig. 1). The patient was disease-free at 4-year follow-up.

## Discussion

Scrotal wall leiomyomas are rare, usually asymptomatic tunica dartos tumours, commonly seen in middle-aged Caucasian men [1]. They are typically slow growing presenting in the fifth decade of life [2].

They are often less than 3 cm in diameter and are more commonly solitary than multiple [3]. The solitary group is further categorised as angioleiomyoma, genital-areolar leiomyoma and piloleiomyoma [3]; it has been suggested that these tumours are myofibroblastic in origin [4]. Typically, these lesions are poorly circumscribed, non-encapsulated tumours consisting of uniform spindle shaped cells arranged as interlacing fascicles with little or no pleomorphism, or mitoses [1,3].

Simple surgical excision is curative; surgery for large lesions should be conservative if its cutaneous origin is clearly separate from the testis or adnexal structures [5]. Radiation should be avoided as it may induce malignant transformation [6]. Recurrence and malignancy have been described [1].

## Conclusion

In symplastic scrotal leiomyoma, the presence of nuclear pleomorphism and mitoses, just falling short of the criteria for malignancy, makes prediction of biological behaviour difficult. Immunohistochemistry helps identify this subgroup of patients who warrant close follow-up in view of the malignant potential.

## Consent

Written informed consent was obtained from the patient for publication of this case report and any accompanying images. A copy of the written consent is available for review by the Editor-in-Chief of this journal.

## Competing interests

The authors declare that they have no competing interests.

## Authors' contributions

JP conceived the case report, collected the patient's information and was involved in writing the manuscript. PV collected the patient's information and was involved in writing the manuscript. RM helped collate patient information and was a major contributor in writing the manuscript. JM conceived the case report with JP, performed the histological examination and was involved in writing the manuscript. All authors read and approved the final manuscript.
